# GAIT AUTOMATICITY AND COMMUNITY MOBILITY OF OLDER ADULTS

**DOI:** 10.1093/geroni/igad104.0548

**Published:** 2023-12-21

**Authors:** Andrea Rosso, Emma Baillargeon, Kyle Moored, Breanna Crane, Michelle Carlson, Theodore Huppert, Jennifer Brach, Caterina Rosano

**Affiliations:** University of Pittsburgh, Pittsburgh, Pennsylvania, United States; University of Pittsburgh, Pittsburgh, Pennsylvania, United States; Johns Hopkins Bloomberg School of Public Health, Baltimore, Maryland, United States; Johns Hopkins Bloomberg School of Public Health, Baltimore, Maryland, United States; Johns Hopkins Bloomberg School of Public Health, Baltimore, Maryland, United States; University of Pittsburgh, Pittsburgh, Pennsylvania, United States; University of Pittsburgh, Pittsburgh, Pennsylvania, United States; University of Pittsburgh, Pittsburgh, Pennsylvania, United States

## Abstract

Gait automaticity may decline with age-related impairments in the brain. As subcortical structures supporting gait automaticity decline, the prefrontal cortex (PFC) may provide compensatory function, allowing for maintenance of mobility but with loss of efficiency. Loss of efficiency may reduce ability to navigate complex community environments and restrict community mobility. We assessed the relation between PFC activation during walking in the laboratory with self-reported and objectively measured community mobility in participants aged 65+ (n=42, mean age=76, 60% female) from a randomized trial of a physical activity intervention to improve walking speed. PFC activation was measured by functional near-infrared spectroscopy as change from quiet standing to usual pace walking. Community mobility was measured objectively by 7-day global positioning system recordings of spatial (standard deviation ellipse area (SDEa), maximum distance from home) and temporal (percent time out of home (pTOH)) characteristics. Additionally, step count was recorded from 7-day actigraphy and the Life-Space Assessment (LSA) assessed self-reported mobility. Among participants with complete data at baseline (n=27), higher PFC activation during walking was associated with smaller SDEa (beta=-0.93 (-1.72, -0.15)), less pTOH (beta=-0.45 (-0.78, -0.12)), and lower step count (beta=-346 (-575, -118)) which persisted after adjusting for age, gender, education, or gait speed. PFC activation was not associated with maximum distance or LSA. There were no significant associations at the post-intervention visits (n=39). Greater PFC activation, likely indicating reduced gait automaticity, is related to lower spatial extent, duration, and intensity of community mobility. This association may be mitigated by participation in physical activity interventions.

